# Indents

**Published:** 1912-03-15

**Authors:** 


### INDENTS.

☞Brief Articles of Technical or Scientific Value will receive notice and comment. Contributions invited.

*Period of Susceptibility to Dental Caries.* We are being more forcibly reminded now than we have ever been before that the period of greatest susceptibility to dental caries is during the period between childhood and puberty. Several reasons have been suggested as explanatory of this fact. We are somewhat unscientific, however, at present, and I will not discuss these reasons. This much we know —and it is not a negative proposition—that all the dental ills from which mankind suffers throughout life are directly due to the neglect of the teeth during a short period in childhood, and if the advertisement of the oral hygiene propaganda succeeds in impressing the profession with this truth, the movement will have been worth while.—*Cosmos.*

*Number of Doctors:* According to Dr. J. K. Scudder (*Eclectic Medical Journal*), there are about 75,000 members of the regular school practising in the United States; 9000 homeopaths; 7500 eclectics; 4000 osteopaths; and 4500 unclassified. Dr. Scudder says that, while the various directories give the names of 120,000 to 130,000 physicians, they contain the names of various men who are deceased, removals, duplicates, and even "undertakers, embalmers, veterinary surgeons, *et al.*"

*Chicago's Tuberculosis Sanitarium.* The city of Chicago has purchased 160 acres of land at North Fortieth, Bryn Mawr, and Peterson avenues, and is expecting to build in this locality a great municipal tuberculosis sanitarium. The plans have been prepared for the buildings and the total cost of the institution is estimated at about $878,000. It will provide accommodations for 15000 patients a year.

